# Longitudinal assessment of natural disease progression in Brazilian children and adolescents with Charcot-Marie-Tooth disease

**DOI:** 10.1055/s-0045-1811174

**Published:** 2025-08-20

**Authors:** Bruno Alvarenga Soares, Camila Fernanda de Freitas, Juliana Cardoso, Cyntia Rogean de Jesus Alves de Baptista, Wilson Marques, Ana Claudia Mattiello-Sverzut

**Affiliations:** 1 Universidade de São Paulo, Faculdade de Medicina de Ribeirão Preto, Ribeirão Preto SP, Brazil.

**Keywords:** Charcot-Marie-Tooth Disease, Functional Status, Disability Evaluation, Child, Adolescent

## Abstract

**Background:**

Charcot-Marie-Tooth disease (CMT) is a progressive neurological disorder that typically manifests in early childhood. The natural progression of CMT in Brazilian pediatric and adolescent populations remains unknown.

**Objective:**

To evaluate the natural disease progression in Brazilian children and adolescents with CMT using the Charcot-Marie-Tooth Pediatric Scale (CMTPedS).

**Methods:**

A longitudinal observational study was conducted to assess disease progression over a 2-year period through 3 evaluations, spaced 1 year apart, in children and adolescents aged 5 to 18 years with a confirmed CMT diagnosis. Height, weight, body mass index (BMI), fat-free mass, and functionality (assessed via the CMTPedS) were evaluated in each of the three sessions.

**Results:**

We included 30 participants of both sexes with a mean age of 11.1 ± 3.2 years. Significant increases in height, weight, and BMI were observed, alongside a decline in the percentage of lean body mass across evaluations. The total score on the CMTPedS increased by 4.5 points throughout 2 years, indicating disease progression, with notable deterioration in functional dexterity, pinprick and vibration sensations, and gait. Significant progression was evident within 1 year, with an average annual deterioration of 2.25 points. The subgroup with CMT type 1A (CMT1A) presented an increase of 4 points in the total score, corresponding to an average annual progression of 2 points. Unlike the overall CMT group, the CMT1 subgroup did not exhibit a decline in the pinprick sensation score.

**Conclusion:**

Children and adolescents with CMT exhibit significant disease progression within 2 years, with measurable deterioration observed as early as 1 year. The CMTPedS is a reliable tool to monitor CMT progression in this population.

## INTRODUCTION

Charcot-Marie-Tooth disease (CMT), one group of peripheral neuropathies, represents one of the most prevalent inherited neurological disorders, with an estimated prevalence of 1 in 1,214 to 2,500 individuals across all sexes and ethnicities. 1 2 3 More than 100 genes have been implicated in the pathogenesis of CMT. 4 While previous large-scale studies 5 6 have demonstrated that most CMT neuropathies are demyelinating, it should be noted that axonal disorders account for up to one-third of the cases. Despite phenotypic variability, most patients present with symptom onset in the first or second decades of life, characterized by distal muscle weakness, sensory loss, and foot deformities. Disease progression is typically slow and lifelong, though some types, such as Dejerine-Sottas neuropathy, manifest with severe, rapidly-progressing disability in early childhood. 7 8

International studies 8 show that CMT progression is generally slow and varies by type, with type 1A (CMT1A, the most prevalent) progressing more slowly than types 1B (CMT1B), 2A (CMT2A), and 4C3 (CMT4C3). Early clinical manifestations, such as atypical neuromotor development and frequent falls, are often reported by parents within the first decade of life. Older children and adolescents typically present with difficulties in running and reduced athletic performance. Additional signs, including distal muscle weakness, sensory deficits, and pes cavus deformity, may also manifest during childhood, facilitating early diagnosis. 9 10 However, there is a need for a scalable tool that can be applied longitudinally to monitor the natural history of the disease. Specific scales, such as the Charcot-Marie-Tooth Disease Pediatric Scale (CMTPedS), 11 are relevant to longitudinally monitor the natural history of CMT based on the composition of items especially related to dysfunctions.

The CMTPedS is a reliable, valid, and sensitive instrument designed for individuals aged 3 to 20 years, providing a comprehensive evaluation of clinical impairment, including strength, dexterity, sensory function, gait, balance, power, and endurance. 11 Its psychometric properties have been rigorously assessed through classical test theory (item, reliability, and factor analysis) and item response theory (Rasch modeling), ensuring its ability to reliably measure disability progression throughout time. 11 The Brazilian version of the CMTPedS has been adapted and validated, 12 enabling a reliable and clinically-relevant assessment of CMT impairment among the Brazilian children and adolescent population.

Most forms of CMT manifest in early childhood, underscoring the importance of understanding the clinical impairments throughout the life cycle. 7 Clinical monitoring of pediatric CMT patients enables the tracking of disease progression, the assessment of motor performance relative to disease severity, and the evaluation of the efficacy of the therapeutic interventions. 8 Additionally, childhood represents an optimal window to initiate treatments before the degeneration of the peripheral nerve worsens and the deformities set in.

Brazil exhibits significant socioeconomic disparities that directly impact education, food security, and, consequently, health-seeking behaviors and treatment access. 13 These inequities—combined with Brazil's heterogeneous healthcare infrastructure, regional variability in rehabilitation services, and unique population genetics—create distinct disease progression modulators, rendering this pediatric investigation both clinically meaningful and methodologically innovative. 14

Whether disease progression in Brazilian children and adolescents with CMT follows the same pattern as in other populations remains unknown. Therefore, the present study aims to evaluate the natural disease progression in children and adolescents with CMT followed at a tertiary hospital in the countryside of the State of São Paulo, Brazil, using the CMTPedS and its subscales.

## METHODS

### Study design

The current is a longitudinal observational study in which volunteers diagnosed with CMT were invited through the Physiotherapy Outpatient Clinic for Children and Adolescents with CMT at the Teaching Hospital of Faculdade de Medicina de Ribeirão Preto, Universidade de São Paulo (USP), Brazil, a tertiary hospital with a reference service for rare diseases that receives patients from all over the country. The legal guardians and the volunteers signed the informed consent form, and the volunteers also signed the informed assent form. The study was approved by the Ethics Committee of USP (under protocol number: 45454620.3.0000.5440).

### Participants

Children and adolescents aged 5 to 18 years were invited to participate. Diagnosis and disease type were confirmed through clinical features, nerve conduction studies and genetic tests. The inclusion criteria were a medical diagnosis of CMT, age between 5 and 18 years, absence of comorbid conditions affecting the lower limbs, no history of fractures, and no recent lower-limb surgery. The exclusion criterium was any acute illness within the 4 weeks preceding evaluations. The participants were enrolled in annual follow-up assessments (with a maximum variation of 2 months) from 2022 to 2024, totaling 3 evaluations. A total of 30 volunteers completed the 1st and 3rd assessments, and 26 volunteers completed the second assessment.

### Procedures

#### *Anthropometric measurements*

Body composition was assessed by measuring height, body mass, and body mass index (BMI). Height was measured using a stadiometer (Wiso, model 210, accuracy of 0.1 cm), with the participants barefoot, standing upright, and looking straight ahead. Body mass was measured using a mechanical scale (Welmy, W200m, accuracy of 0.1 kg), with the participants wearing light clothing. The BMI was calculated as body mass (kg) divided by height squared (m ^2^ ).

The percentage of fat-free mass was evaluated using bioelectrical impedance (Biodynamics, BIA 450), with the participants in a supine position and sensor pads placed on the right wrist-hand and ankle-foot according to manufacturer guidelines.

#### *Charcot-Marie-Tooth Disease Pediatric Scale*

The CMTPedS is a standardized clinical outcome measure, validated for Brazilian Portuguese, comprising 11 performance-based assessments: the functional dexterity test, the nine-hole peg test, the hand grip strength, foot dorsiflexion and plantarflexion strength (measured using hand-held dynamometry), pinprick and vibration sensations, balance, gait, long jump, and the six-minute walk test (6MWT). 11 12 The total score ranges from 0 to 44 points, with higher scores indicating greater disease severity. Disease severity is classified as mild (0–14 points), moderate (15–29 points), or severe (30–44 points). 5

Vibration sensation testing required the use of a Rydel-Seiffer tuning fork with a frequency of C 64 Hz/c 128 Hz and detachable clamps (Martin). The procedures followed the protocol of previous CMTPedS studies. 11 12 15

### Statistical analysis

Data distribution was assessed using the Shapiro-Wilk test for normality. Descriptive statistics were reported as mean and standard deviation values. One-way repeated measures analysis of variance (ANOVA) was used to compare the mean values obtained across the three annual evaluations. The assumption of sphericity was evaluated using the Mauchly's test. If the test indicated a violation of sphericity ( *p* < 0.05), adjustments to the degrees of freedom (Greenhouse-Geisser correction) were applied to ensure the validity of the analysis. Post-hoc analyses with Bonferroni correction were conducted to identify specific time points in which significant differences occurred. The results were reported as mean and 95%CI values, with the F-value (the ratio of between-group variance to within-group variance) and the corresponding degrees of freedom for both between-group and within-group effects. A significance level of 0.05 was applied to all statistical tests. Data analysis was performed using IBM SPSS Statistics for Windows (IBM Corp.) software, version 29.0.

Power sample analysis was performed a posteriori using the G*Power 3.1.9.7 software (free), with the variance values derived from the total CMTPedS scores of our 30 study participants. The power sample was of 0.99 (99%).

## RESULTS

The current study included 30 volunteers diagnosed with CMT, of both sexes (and 56.3% of female subjects), with a mean age of 11.1 ± 3.2 years. Among the participants, 24 were diagnosed with CMT1A, 4, with CMT2A, 1, with CMT1B, and 1, with CMT2K. The demographic and clinical characteristics of the volunteers are detailed in Table 1 .

**Table 1:** Characteristics of the volunteers in the 3 evaluations of the study in mean and 95%CI values

	Evaluation 1 ( *n* = 30)	Evaluation 2 ( *n* = 26)	Evaluation 3 ( *n* = 30)	*p*
Height (cm)	150.1 (144.0–156.3)	153.3 (147.6–158.9) #	155.7 (151.0–160.1) #$	**< 0.0001**
Weight (Kg)	48.3 (40.6–56.0)	51.4 (42.9–59.1) #	54.3 (46.1–61.6) #$	**< 0.0001**
Body Mass Index (kg/m ^2^ )	20.4 (17.8–21.3)	21.0 (19.3–24.7)	22.0 (20.2–25.5) #	**0.033**
Fat-free mass (%)	75.1 (66.2–84.0)	66.8 (60.8–79.9)	62.0 (56.3–67.7) #	**0.039**
Walker (n)	29	25	29	**-**
Wheelchair user (n)	1	1	1	**-**
Previous surgeries (n)	0	0	0	**-**
Rehabilitation (n)	7	6	10	**-**
Physical activity (n)	0	7	8	**-**
Other therapies (n)	0	0	0	**-**
Orthosis (n)	5	6	11	**-**
Insole (n)	3	6	4	**-**

Notes: #Significant difference from evaluation 1; $significant difference from evaluation 2.

There was a significant difference in terms of height in the 3 evaluations (F [1.234–27.146] = 12.417; *p* < 0.0001), with evaluation 1 versus evaluation 2 with *p* = 0.005, evaluation 1 versus evaluation 3 with *p* = 0.004, and evaluation 2 versus evaluation 3 with *p* = 0.03. The same results were found regarding weight (F [1.490–32.778] = 21.332; *p* < 0.0001), with evaluation 1 versus evaluation 2 with *p* = 0.004, evaluation 1 versus evaluation 3 with *p* < 0.0001, and evaluation 2 versus evaluation 3 with *p* = 0.001. Regarding the BMI (F [1.511–24.179] = 4.575; *p* = 0.033), there was a significant difference only between evaluations 1 and 3 ( *p* = 0.03), with evaluation 1 versus evaluation 2 with *p* = 0.59, and evaluation 2 versus evaluation 3 with *p* = 0.59. In terms of the percentage of fat-free mass (F [1.875–5.624] = 2.765; *p* = 0.039), there was significant difference only between evaluations 1 and 3 ( *p* = 0.04), with evaluation 1 versus evaluation 2 with *p* = 0.23, and evaluation 2 versus evaluation 3 with *p* = 0.48.

The scores on the CMTPedS are shown in Table 2 . There was significant difference regarding the total score in the 3 evaluations (F [1.487–37.178] = 12.467; *p* < 0.0001), with evaluation 1 versus evaluation 2 with *p* = 0.001, evaluation 1 versus evaluation 3 with *p* = 0.001, and evaluation 2 versus evaluation 3 with *p* = 0.001.

**Table 2:** Scores on the Charcot-Marie-Tooth Disease Pediatric Scale in the 3 evaluations in mean and 95%CI values

	Evaluation 1 ( *n* = 30)	Evaluation 2 ( *n* = 26)	Evaluation 3 ( *n* = 30)	*p*
Functional dexterity test	1.7 (1.1–2.3)	2.5 (1.9–3.1) #	2.6 (2.0–3.1) #	**0.007**
Nine-hole peg test	3.0 (2.5–3.4)	3.0 (2.5–3.4)	3.2 (2.8–3.6)	0.52
Hand grip strength	1.1 (0.5–1.8)	1.2 (0.5–1.8)	0.8 (0.2–1.4)	0.28
Dorsiflexor strength	2.6 (2.1–3.1)	2.9 (2.3–3.3)	2.9 (2.3–3.3)	0.52
Plantar flexor strength	2.4 (1.8–2.9)	2.7 (2.2–3.2)	2.5 (1.8–3.0)	0.50
Pinprick sensation	0.4 (0.0–0.8)	0.7 (0.2–1.2)	1.4 (0.8–1.8) #	**0.019**
Vibration sensation	0.5 (0.1–0.9)	0.6 (0.1–1.1)	1.5 (0.8–2.0) #$	**0.004**
Balance	1.8 (1.1–2.5)	1.8 (1.0–2.4)	2.0 (1.3–2.7)	0.59
Gait	1.9 (1.4–2.4)	2.4 (1.9–2.8)	2.8 (2.2–3.1) #	**0.008**
Long jump	2.4 (1.9–2.9)	2.5 (2.0–3.0)	2.5 (2.0–2.9)	0.89
6-minute walk test	2.5 (2.0–2.9)	2.7 (2.1–3.1)	2.7 (2.2–3.1)	0.63
Total score	20.1 (17.0–23.2)	22.6 (19.4–25.8) #	24.6 (21.1–28.2) #$	**< 0.0001**

Notes: #Significant difference from evaluation 1; $significant difference from evaluation 2.

Moreover, there was significant difference regarding the functional dexterity test (F [1.915–47.884] = 4.139; *p* = 0.007), with a significant difference only between evaluations 1 and 3 ( *p* = 0.018), and evaluation 1 versus evaluation 2 with *p* = 0.027, and evaluation 2 versus evaluation 3 with *p* = 0.89. In the pinprick item (F [1.663–39.920] = 7.451; *p* = 0.019), the was a significant difference only between evaluations 1 and 3 ( *p* = 0.01), with evaluation 1 versus evaluation 2 with *p* = 0.6, and evaluation 2 versus evaluation 3 with *p* = 0.32.

Regarding the vibration sensation (F [1.889–47.481] = 6.355; *p* = 0.004), there was a significant difference between evaluations 1 and 3 ( *p* = 0.01), with evaluation 2 versus evaluation 3 with *p* = 0.041, and evaluation 1 versus evaluation 2 with *p* = 0.98.

In terms of the gait scores (F [1.844–42.402] = 5.682; *p* = 0.008), there was a significant difference only between evaluations 1 and 3 ( *p* = 0.01), with evaluation 1 versus evaluation 2 with *p* = 0.12, and evaluation 2 versus evaluation 3 with *p* = 0.62).

There was no significant difference in the following subitems: nine-hole peg test (F [1.702–42.556] = 0.611; *p* = 0.52; with evaluation 1 versus evaluation 2 with *p* = 1, evaluation 1 versus evaluation 3 with *p* = 1, and evaluation 2 versus evaluation 3 with *p* = 1); hand grip strength (F [1.915–47.884] = 1.279; *p* = 0.28; with evaluation 1 versus evaluation 2 with *p* = 1, evaluation 1 versus evaluation 3 with *p* = 0.34, and evaluation 2 versus evaluation 3 with *p* = 0.6); dorsiflexor strength (F [1.683–42.086] = 0.596; *p* = 0.52; with evaluation 1 versus evaluation 2 with *p* = 0.69, evaluation 1 versus evaluation 3 with *p* = 1, and evaluation 2 versus evaluation 3 with *p* = 1); plantar flexor strength (F [1.802–45.051] = 0.596; *p* = 0.5; with evaluation 1 versus evaluation 2 with *p* = 1, evaluation 1 versus evaluation 3 with *p* = 1, and evaluation 2 versus evaluation 3 with *p* = 1); balance (F [1.804–45.110] = 0.497; *p* = 0.59; with evaluation 1 versus evaluation 2 with *p* = 0.12, evaluation 1 versus evaluation 3 with *p* = 0.019, and evaluation 2 versus evaluation 3 with *p* = 0.62); long jump (F [1.890–47.238] = 0.100; *p* = 0.89; with evaluation 1 versus evaluation 2 with *p* = 1, evaluation 1 versus evaluation 3 with *p* = 1, and evaluation 2 versus evaluation 3 with *p* = 1); and 6MWT (F [1.763–44.064] = 0.427; *p* = 0.63; with evaluation 1 versus evaluation 2 with *p* = 1, evaluation 1 versus evaluation 3 with *p* = 1, and evaluation 2 versus evaluation 3 with *p* = 1).

In the analysis restricted to the volunteers diagnosed with CMT1A ( Table 3 ), a significant difference was observed in the total CMTPedS score (F [1.462–29.245] = 8.140; *p* = 0.004). The post-hoc comparisons revealed significant differences between evaluations 1 and 3 ( *p* = 0.012) and evaluations 1 and 2 ( *p* = 0.003), but not between evaluations 2 and 3 ( *p* = 0.63).

**Table 3:** Scores on the Charcot-Marie-Tooth Disease Pediatric Scale in the 3 evaluations in mean and 95%CI values regarding the CMT1A volunteers

	Evaluation 1 ( *n* = 24)	Evaluation 2 ( *n* = 22)	Evaluation 3 ( *n* = 24)	*p*
Functional dexterity test	1.7 (1.0–2.4)	2.3 (1.6–3.0)#	2.4 (1.8–3.1)#	**0.027**
Nine-hole peg test	2.9 (2.4–3.3)	3.0 (2.4–3.5)	3.1 (2.7–3.5)	0.63
Hand grip strength	0.7 (0.1–1.2)	0.8 (0.1–1.4)	0.6 (0.2–1.0)	0.49
Dorsiflexor strength	2.3 (1.8–2.8)	2.6 (2.0–3.1)	2.7 (2.1–3.3)	0.52
Plantar flexor strength	2.1 (1.5–2.7)	2.6 (2.0–3.2)	2.3 (1.5–2.8)	0.28
Pinprick sensation	0.3 (0.0–0.7)	0.6 (0.1–1.0)	1.0 (0.5–1.5)	0.15
Vibration sensation	0.5 (0.1–1.0)	0.6 (0.1–1.2)	1.3 (0.7–1.9)#$	**0.029**
Balance	1.5 (0.8–2.3)	1.4 (0.6–2.1)	1.9 (1.1–2.7)	0.18
Gait	1.8 (1.3–2.3)	2.3 (1.7–2.7)	2.5 (1.9–3.0)#	**0.029**
Long jump	2.2 (1.7–2.8)	2.3 (1.9–2.8)	2.3 (1.8–2.7)	0.87
6-minute walk test	2.3 (1.7–2.7)	2.6 (2.0–3.1)	2.5 (1.9–3.0)	0.53
Total score	18.3 (15.4–21.7)	21.0 (18.1–23.8)#	22.3 (19.0–25.7)#	**0.004**

Abbreviation: CMT1A, Charcot-Marie-Tooth disease type 1A.

Notes: #Significant difference from evaluation 1; $significant difference from evaluation 2.

Similarly, the functional dexterity test showed a significant difference (F [1.879–37.581] = 2.278; *p* = 0.027), with the post-hoc tests indicating significant differences between evaluations 1 and 3 ( *p* = 0.049) and evaluations 1 and 2 ( *p* = 0.045), but not between evaluations 2 and 3 ( *p* = 0.93). For vibration sensation, a significant effect was observed (F [1.960–39.195] = 3.868; *p* = 0.029), with the post-hoc comparisons revealing differences between evaluations 1 and 3 ( *p* = 0.043) and evaluations 2 and 3 ( *p* = 0.049), but not between evaluations 1 and 2 ( *p* = 0.99). Moreover, gait scores exhibited a significant difference (F [1.999–35.997] = 3.915; *p* = 0.029), with the post-hoc tests indicating a significant difference only between evaluations 1 and 3 ( *p* = 0.048), but not between evaluations 1 and 2 ( *p* = 0.21) or evaluations 2 and 3 ( *p* = 0.76).

In contrast to previous analyses including all volunteers, the pinprick sensation item showed no significant differences across the three evaluations in the CMT1A subgroup. The remaining items exhibited no significant differences, which is consistent with the findings from the full analyses with all volunteers.

## DISCUSSION

The current research assessed disease progression over a 2-year period through 2 evaluations. The findings showed an increase in height, weight, and BMI, alongside a reduction in lean body mass percentage among the volunteers. Regarding the CMTPedS, a deterioration in the total score was observed, as well as in the scores on the subitems of the functional dexterity test, pinprick sensation, vibration sensation, and gait. In contrast to the analyses involving all participants, the pinprick sensation item showed no significant differences across the three evaluations in the CMT1A subgroup.

Weight and height increased over the 2-year period of evaluation, as expected in children and adolescents due to the developmental stage. 16 17 However, the BMI also increased. Although the rise in BMI did not indicate overweight status, 18 this finding should be monitored closely to enable potential interventions if the BMI continues to rise. In a longitudinal study 19 on the association of BMI with disease progression in 242 children and adolescents with CMT aged 3 to 20 years, those who were severely underweight, underweight, or obese presented greater baseline disability. Over the 2-year period, severely-underweight children with stable BMIs presented the most rapid deterioration. 19 Among participants who changed BMI categories, the CMTPedS scores declined faster in those who transitioned to overweight/obese status. Interventions targeting the maintenance or improvement of BMI within a healthy range may mitigate disability progression in children with CMT. 19

However, regarding the percentage of fat-free mass, a reduction was observed, accompanied by a corresponding increase in adipose tissue. In healthy children and adolescents, the average fat-free mass ranges from 89.99 to 80% in boys aged 7 to 17 years and from 84.99 to 75% in girls aged 7 to 17 years, 20 indicating that children and adolescents with CMT exhibit values significantly below the expected range for their age through a short period of time. Nutritional monitoring and the promotion of physical exercise should be encouraged in this population, given the significant adverse effects that overweightness and obesity can cause, such as cardiovascular and metabolic diseases. 21 The current study did not assess sedentary behavior among the participants, highlighting the need for further investigation of these factors in future studies.

Additionally, disease progression in CMT leads to progressive muscle tissue loss, reducing lean mass. 5 19 When combined with potential sedentary behaviors due to physical activity barriers, this accelerates unfavorable body composition changes (increased adiposity). 19 22 Notably, though most participants had CMT1A (which is typically milder), they still presented a significant deterioration in the CMTPedS score—underscoring the need for longitudinal Brazilian cohort studies tracking all CMTPedS domains (not just those showing acute decline) in larger samples.

Ideally, patients should receive lifelong monitoring starting at early diagnosis. However, Brazil's continental dimensions and regional variability in access to healthcare services, combined with sociocultural factors, may influence the timing and quality of clinical care. 14 The divergent findings regarding the present Brazilian study and previous international research in developed countries may reflect such heterogeneity. Children with CMT in these settings are more likely to benefit from early diagnosis and comprehensive multidisciplinary care—advantages that may still be limited in some regions of Brazil due to the uneven distribution of resources.

In the current study, the total score on the CMTPedS showed a significant difference as early as 1 year, with a more pronounced deterioration in the following year. Over the 2-year period, there was an increase of 4.5 points in the total score, corresponding to an average annual deterioration of 2.25 points. The CMT1A subgroup presented an increase of 4 points in the total score, reflecting an average annual progression of 2 points—slower than that observed in the overall CMT group. These findings differ from the values reported in studies conducted in other countries. 8 In a longitudinal study with 15 children (aged 4–17 years) with mixed CMT genetic types, disease progression was observed at a rate of 1.0 CMTPedS point per year, representing a 5% change from baseline. 11 Moreover, a cross-sectional study 5 with 520 CMT patients further revealed that the total CMTPedS scores progressed consistently throughout early childhood (ages 3–10 years) and adolescence (ages 11–20 years) in CMT1A, whereas the rate of change in CMT1B, CMT2A, and CMT4C appeared to be age-specific. Additionally, the CMTPedS demonstrated greater sensitivity to change compared with the Rasch-weighted Charcot-Marie-Tooth Neuropathy Score (CMTNS) in older children. 5

A multicentric longitudinal study 8 involving 206 (103 female) participants aged 3 to 20 years assessed disease progression over a 2-year period using the CMTPedS, revealing an overall progression rate of 2.4 points (14%) across all types. Children with CMT1A, the most common type, progressed at a rate of 1.8 points (12%), while those with CMT2A and CMT4C presented faster progression rates compared with CMT1A, and progression in CMT1B was highly variable. 8 Similar results were found with children and adolescents with CMT1A in the present study when they were analyzed separately from the other CMT subtypes.

Although the CMTPedS scores in the current study demonstrated a significant increase through a short period of time, there was no change in the disease severity classification, with participants consistently presenting a moderate severity level, even when analyzing CMT subtypes together and the CMT1A subtype separately. 5 These volunteers should be monitored over an extended period to enhance our understanding of the natural progression of the disease. Additionally, it is important to consider that participants may receive interventions, such as orthoses or other assistive devices, aimed at improving functionality.

In a Brazilian study 23 involving children and adolescents with CMT aged 7 to 17 years, no significant differences were observed in anthropometric measures, range of motion, dynamometric assessments (inversion, eversion, plantar flexors, dorsiflexors, knee extensors, hip extensors), foot alignment, long jump, balance, or the 10-meter walk test over a 6-month follow-up period. These findings suggest that disease progression may only become evident after this initial period, as observed in the current study.

Regarding the individual items of the CMTPedS in all volunteers with CMT in the present study, only the functional dexterity test, pinprick sensation, vibration sensation, and gait demonstrated significant deterioration compared with the initial assessment. These findings contrast with those of the multicenter longitudinal study, 8 which reported progression in all individual items of the CMTPedS over a 2-year period. In that study, 8 the most responsive items were foot dorsiflexion strength, balance, and long jump, differing from the present study, in which these items did not exhibit a significant decline. The absence of significant alterations in pinprick sensation among the CMT1A subgroup over the 2-year period aligns with the literature, 8 as this subtype typically presents a milder phenotype compared with other subtypes. These findings highlight the heterogeneity in disease progression among CMT types and demonstrate the usefulness of the CMTPedS in monitoring functional decline in pediatric patients. In the aforementioned study, 8 58% of the participants were diagnosed with CMT1A, compared with 80% in the current study. Moreover, the mean age was higher in the current study (11.1 ± 3.2 years versus 9.8 ± 3.9 years in the aforementioned study), 8 which may complicate the interpretation of these discrepant results. Given that CMT is a progressive disease, interventions aimed at maintaining or improving functionality are often implemented from the initial diagnosis and continue throughout the disease course. Lower limb functional impairments, such as gait disturbances and foot deformities, are typically the first symptoms recognized by both parents and healthcare providers. This early recognition leads to rehabilitation (e.g., orthoses, gait devices, corrective surgeries) being prioritized a focus that may explain why functional decline was less pronounced in these areas compared with fine motor skills. 8 23 24 25 Notably, the total CMTPedS score demonstrated significant worsening in terms of the pinprick sensation across all patients. However, the absence of significant changes in pinprick sensation within the CMT1A subgroup over the 2-year period is consistent with the literature, 8 as this subtype typically exhibits a milder phenotype compared with others. The stability of other subitems during the observation interval may reflect compensatory mechanisms preserving function in these areas, warranting further mechanistic investigation.

Finally, a previous study 8 examining the natural progression of CMT in children and adolescents with assessments at baseline and a 2-year (± 6 months) follow-up conducted a sensitivity analysis using analysis of covariance (ANCOVA) to observe if the ± 6-month evaluation interval variation between volunteers' assessments could be a covariance and interfere in the results of the study, but it found no significant differences. In contrast, the present study maintained a stricter evaluation window (maximum variation of 2 months between annual assessments), which may be considered a methodological strength.

The current study has some limitations, including the relatively mild disease severity of the CMT population studied, which consisted predominantly of CMT1A cases, which may restrict the generalizability of the findings to children with more advanced disease stages, as well as the small sample size. Even with the hospital receiving patients from different regions of the country, a multicenter study could provide a broader representation of disease profiles and enhance sample diversity. Additionally, since our rehabilitation center is part of a tertiary hospital, the participants did not necessarily undergo rehabilitation treatment within our facility. As a result, it was not possible to monitor the type or intensity of interventions they received during the study period. Therefore, we cannot rule out the possibility that external treatments may have influenced the outcomes observed. Furthermore, while longer-term monitoring of these volunteers would be valuable, the CMTPedS is only validated for individuals up to 20 years of age.

Children and adolescents with CMT present significant disease progression over a 2-year period, with measurable deterioration evident as early as 1 year. The CMTPedS has proven to be a reliable tool to monitor CMT progression in this population.
